# Balanced Biochemical Reactions: A New Approach to Unify Chemical and Biochemical Thermodynamics

**DOI:** 10.1371/journal.pone.0029529

**Published:** 2012-01-11

**Authors:** Antonio Sabatini, Alberto Vacca, Stefano Iotti

**Affiliations:** 1 Dipartimento di Chimica, Università di Firenze, Sesto Fiorentino, Italy; 2 Dipartimento di Medicina Interna dell'Invecchiamento e Malattie Nefrologiche, Università di Bologna, Bologna, Italy; 3 National Institute of Biostructures and Biosystems, Rome, Italy; University of Groningen, The Netherlands

## Abstract

A novel procedure is presented which, by balancing elements and electric charge of biochemical reactions which occur at constant pH and pMg, allows assessing the thermodynamics properties of reaction Δ*_r_G* ^′0^, Δ*_r_H* ^′0^, Δ*_r_S* ^′0^ and the change in binding of hydrogen and magnesium ions of these reactions. This procedure of general applicability avoids the complex calculations required by the use of the Legendre transformed thermodynamic properties of formation Δ*_f_G* ^′0^, Δ*_f_H* ^′0^ and Δ*_f_S* ^′0^ hitherto considered an obligatory prerequisite to deal with the thermodynamics of biochemical reactions. As a consequence, the term “conditional” is proposed in substitution of “Legendre transformed” to indicate these thermodynamics properties. It is also shown that the thermodynamic potential *G* is fully adequate to give a criterion of spontaneous chemical change for all biochemical reactions and then that the use of the Legendre transformed *G*′ is unnecessary. The procedure proposed can be applied to any biochemical reaction, making possible to re-unify the two worlds of chemical and biochemical thermodynamics, which so far have been treated separately.

## Introduction

Two types of equations are used to describe chemical and biochemical reactions. Chemical equations are written in terms of specific ionic and molecular species and balance elements and charge, biochemical equations are written in terms of reactants (sum of species) that consist of species in equilibrium with each other and do not balance elements that are assumed to be fixed, such as hydrogen and magnesium at constant pH and pMg [1]. Therefore, any biochemical reaction defines a sub-system of chemical reactions involving the complex species formed by the ligands (the poly-anionic molecules involved in biological metabolism) and the Lewis acids (H^+^ and the metal ions present in the cell such as Mg^2+^, Na^+^ and K^+^). Both types of reaction have corresponding equilibrium constant: *K* for chemical reaction and *K*′ for biochemical reaction. The conditional equilibrium constant *K*′ (also known as apparent constant) for a biochemical reaction is written in terms of sum of species [2].

The IUBMB-IUPAC Joint Commission on Biochemical Nomenclature (JCBN) states: “When pH and pMg are specified, a whole new set of transformed thermodynamic properties come into play. These properties are different from the usual Gibbs energy *G*, enthalpy *H* and entropy *S*, …, and they are referred to as the transformed Gibbs energy *G′*, transformed enthalpy *H*′, transformed entropy *S′*, …” [1]. Moreover, Alberty [3] states that when pH and pMg are held constant the transformed Gibbs energy *G*′, and not *G*, is minimized at equilibrium and therefore *G*′, and not *G*, gives the criterion of spontaneous chemical change for a biochemical reaction. However, this has been postulated but not demonstrated and on the basis of this assumption the concept that chemical and biochemical thermodynamics are two separate worlds took place [3]–[7] and it was adopted by the IUBMB-IUPAC (JCBN). As a consequence, the existence of two categories of thermodynamics based on different concepts and different formalisms is now established: i) the chemical thermodynamics which makes use of the conventional thermodynamic properties and it is suitable to deal with only chemical reactions; ii) the biochemical thermodynamics which makes use of “unconventional” (transformed) thermodynamic properties and it is suitable to deal with only biochemical reactions.

However, the Legendre transformed Gibbs energy change for a biochemical reaction, Δ*_r_G*′, has been recently shown to be equal to the non-transformed Gibbs energy change, Δ*_r_G*, of any single reaction involving selected chemical species of the biochemical system [8]. These two Gibbs energies of reaction had theretofore been thought to have different values [1], [7], [9]–[12]. The equality Δ*_r_G*′ = Δ*_r_G* does not apply to enthalpy or entropy changes. According to this, the calculation of the transformed enthalpy and entropy changes Δ*_r_H*′ and Δ*_r_S*′ of a biochemical reaction must still be performed solely by the use of the standard transformed enthalpy and entropy of formation [8].

The aim of this study is to show that the procedure of balancing the biochemical reactions makes possible to treat them as chemical reactions avoiding the use of the complicated transformed thermodynamic properties. This approach allows assessing Δ*_r_G* ^′0^, Δ*_r_H* ^′0^, Δ*_r_S* ^′0^ and the change in binding of hydrogen and magnesium ions of any biochemical reaction using the conventional thermodynamic properties of species at various *T*, *P*, pH, pMg, and ionic strength *I*. The presented approach is of general applicability and therefore all the biochemical reactions can be treated by this procedure. This means the two worlds of chemical and biochemical thermodynamics, which so far have been treated separately, can be completely re-unified within the same thermodynamic framework.

## Methods

### Balanced biochemical reactions

Let us consider a general biochemical reaction
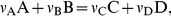
(1)where ν_A_, ν_B_, ν_C_ and ν_D_ are the stoichiometric coefficients. The biochemical reactants A, B, C , D are formed by *N*_A_ species A*_i_*, *N*_B_ species B*_i_*, *N*_C_ species C*_i_* and *N*_D_ species D*_i_* respectively.

**Figure 1:**
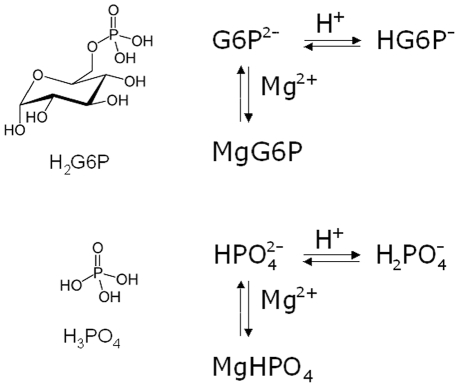
Chemical equilibria between the biochemical reactants of glucose-6-phosphate hydrolysis and H^+^ and Mg^2+^.

We take as example for our exposition the hydrolysis reaction of glucose-6-phosphate. The chemical reaction is
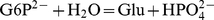
(2)and the biochemical reaction is
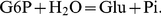
(3)G6P represents the sum of the free species G6P^2−^ and of all the complex species formed by G6P^2−^ with the Lewis's acids H^+^ and Mg^2+^; Glu stands for glucose; Pi represents the sum of the free species 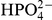 and of all the complex species formed by 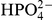 with the Lewis acids H^+^ and Mg^2+^. In the pH and pMg ranges present in the cell cytosol matrix (pH≈7 and pMg≈3.5) the complex species of G6P^2−^ and 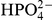 are: HG6P^−^, MgG6P, 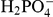 and MgHPO_4_. A scheme of such equilibria is shown in Fig. 1. Here and hereafter pH and pMg values are concentration based, i.e. pH = −log[H^+^] and pMg = −log[Mg^2+^].

The following assumptions are made:

1. free and complex species of each biochemical reactant are at equilibrium with each other;
2. water activity is constant;
3. temperature *T*, pressure *P*, and ionic strength *I* of the solution are constant;
4. pH and pMg are constant;
5. standard concentration *c* ^0^ is 1 M.

The procedure of assessing the change in standard Gibbs energy, in standard enthalpy, in standard entropy and in binding of hydrogen and magnesium ions of a biochemical reaction, starts from balancing elements and charge. The steps needed to balance the biochemical reaction are the following:

1. the concentrations of all the free and complex species at equilibrium have to be calculated by solving the mass balance equations for standard concentration *c* ^0^ of the biochemical reactants at given pH and pMg;
2. the stoichiometric coefficient of each species is equal to its own concentration multiplied by the stoichiometric coefficient of the corresponding biochemical reactant;
3. the stoichiometric coefficients of Mg^2+^ and H^+^ are obtained by balancing the atoms of Mg and H in both terms of the reaction.

The correctness of the whole procedure may be verified by checking that the total ionic charge is the same in both terms of the reaction.

Step 1 of the procedure for balancing the biochemical reaction is based on the calculation of the concentration of free and complex species in a 1 M solution of the biochemical reactants at specified pH and pMg. Almost all biochemical reactions have complex species that are mononuclear with respect to each reactant. In this case the mass balance equation of each biochemical reactant is easily solved using binding polynomials [13] and the free and complex species concentration are obtained [8]. More generally, in case of poly-nuclear complexation the mass balance equations can be solved using any of the available computer speciation programs, like Hyss [14], therefore the general validity of the procedure is maintained. It is to be pointed out that the sum of the concentrations of the species forming each biochemical reactant is equal to *c* ^0^. The ratio between the concentration *c_i_* of the *i*-th species and the concentration *c* ^0^ of the biochemical reactant will be referred as fractional population of species *i* and indicated as *f_i_*∶ *f_i_* = *c_i_*/*c* ^0^.

In step 2, the stoichiometric coefficients of the balanced biochemical reaction are obtained multiplying the concentration of each species by the stoichiometric coefficient of the corresponding biochemical reactant:
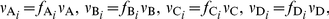
(4)where 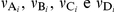 are the stoichiometric coefficients of the species A*_i_*, B*_i_*, C*_i_* and D*_i_*, respectively. Referring to the general biochemical equation (1), we have
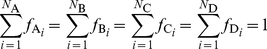
(5)

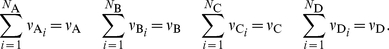
(6)As a consequence, for the balanced biochemical reaction corresponding to the biochemical reaction (3), the following equations hold:
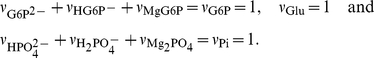
(7)


In step 3 the Mg and H atoms have to be balanced. Taking the stoichiometric coefficients negative for reactants and positive for products and indicating with 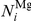 and 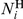 the number of atoms of Mg and H contained in reactant *i*, the Mg^2+^ and H^+^ stoichiometric coefficients are
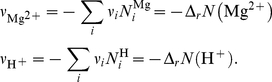
(8)Referring to the Aberty's scheme of the reaction chamber connected to semi-permeable membranes [5], the changes in binding Δ*_r_N*(H^+^) and Δ*_r_N*(Mg^2+^) represent, respectively, the amount of ions H^+^ and Mg^2+^ entering or exiting (depending if they are reactants or products) from the reaction chamber through semi-permeable membranes. It must be underlined that the balanced biochemical reaction so obtained represents the actual transformation processes taking place in the cytosolic solution when the reactants, at a given pH and pMg, transform into products.

The calculations of the stoichiometric coefficients are detailed in Supporting Information S1. The balanced biochemical reaction (*T* = 298.15 K, *I* = 0.25 M, pH = 7, pMg = 3) is:
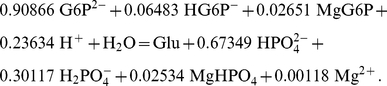
(9)It must be noted that the stoichiometric coefficients of biochemical reactions are usually fractional numbers, as compared to integer coefficients of chemical reactions. The change in the binding of hydrogen and magnesium ions is a direct result of the balancing of the reaction (see Supporting Information S1 and Eq. (S1–14)). The procedure here proposed is of general validity and can be applied to any biochemical reaction with no restriction (see Supporting Information S1 for more details).

## Results and Discussion

### Calculation of Δ_r_H^′0^, Δ_r_G^′0^ and Δ_r_S^′0^

The conditional standard enthalpy change Δ*_r_H* ^′0^ of the biochemical reaction can now be calculated as a sum of the products of the stoichiometric coefficient of each reactant species and its standard enthalpy of formation:
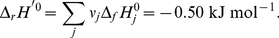
(10)This value matches with that obtained by Alberty [4]. The values of the terms in the summation of Eq. (10) are reported in Table 1 (fifth column). It is worth to underline that this procedure does not require the computation of the transformed standard enthalpy of formation of reactants and products.

**Table 1:** Calculation of Δ*_r_H* ^′0^ and Δ*_r_G* ^′0^ for the hydrolysis of G6P.

Species	Δ*_f_ H* ^0^ a (kJ mol^−1^)	Δ*_f_ G* ^0^ a (kJ mol^−1^)	ν^b^	νΔ*_f_ H* ^0^ (kJ mol^−1^)	*c_i_*	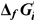 (kJ mol^−1^)	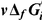 (kJ mol^−1^)
G6P^2−^	−2274.80	−1767.18	−0.90866	2067.02	0.90866	−1767.42	1605.98
HG6P^−^	−2274.23	−1801.40	−0.06483	147.43	0.06483	−1808.18	117.122
MgG6P	−2732.04	−2234.08	−0.02651	72.43	0.02651	−2243.08	59.47
H_2_O	−285.83	−237.19	−1.00000	285.83	1	−237.19	237.19
Glu	−1262.19	−915.90	1.00000	−1262.19	1	−915.90	−915.90
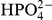	−1297.36	−1099.34	0.67349	−873.76	0.67349	−1100.32	−741.06
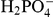	−1302.19	−1138.11	0.30117	−392.18	0.30117	−1141.08	−343.66
MgHPO_4_	−1753.80	−1566.87	0.02534	−44.43	0.02534	−1575.98	−39.93
Mg^2+^	−456.36	−458.54	0.00118	−0.55	1.0·10^−3^	−475.66	−0.56
H^+^	0.41	−0.81	−0.23634	−0.10	1.0·10^−7^	−40.77	9.63

				Δ*_r_H* ^′0^ c (kJ mol^−1^)			Δ*_r_G* ^′0^ d (kJ mol^−1^)
				−0.50			−11.61

a Values from reference [4].

b Stoichiometric coefficients are negative for reactants and positive for products.

c Calculated by adding the terms of column 5.

d Calculated by adding the terms of column 8.

The conditional standard Δ*_r_G* ^′0^ value of the generic biochemical reaction (1) is the change in Gibbs energy when the reagents (sum of species) form the products (sum of species) all in the standard state of concentration *c* ^0^. The concentration *c_i_* of each species is equal to its fractional population *f_i_* times the concentration *c* ^0^ of the biochemical reactant: *c_i_* = *f_i_ c* ^0^. The conditional Gibbs energy of formation of species *i* at concentration *c_i_*, indicated as 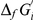, is:
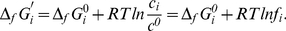
(11)The concentrations of H^+^ and Mg^2+^ are respectively 10^−pH^ and 10^−pMg^ and therefore
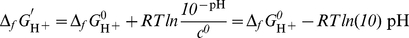
(12)

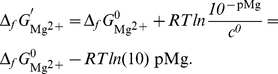
(13)The fractional population of H_2_O is equal to one and then
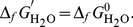
(14)The Δ*_r_G* ^′0^ value of the biochemical reaction is
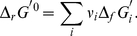
(15)The values of Gibbs energy of formation Δ*_f_G*′ of the reactants of the balanced biochemical reaction (9) are calculated by Eqs. (11–14). Substituting them into (15) we obtain Δ*_r_G* ^′0^ = −11.61 kJ mol^−1^ which perfectly agrees with the value reported by Alberty [4]. The values of the terms in the summation (15) are reported in the last column of Table 1. Therefore, the complex computations required to obtain the standard transformed Gibbs energy of formation are unnecessary.

The value of Δ*_r_S* ^′0^ can be calculated: Δ*_r_S* ^′0^ = (Δ*_r_H* ^′0^−Δ*_r_G* ^′0^)/*T*. However, Δ*_r_S* ^′0^ may also be obtained directly by Δ*_f_S* ^0^of reactants of the balanced biochemical reaction. It must be underlined that reactants are not at standard concentration and therefore the Δ*_f_S* ^0^ values have to be corrected for the entropy of dilution:
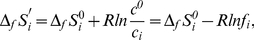
(16)

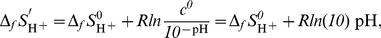
(17)

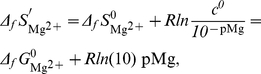
(18)

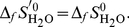
(19)The Δ*_r_S* ^′0^ of the biochemical reaction is:
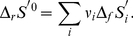
(20)


The balanced biochemical equation allows to obtain the values of Δ*_r_N*(H^+^), Δ*_r_N*(Mg^2+^), Δ*_r_H* ^′0^, Δ*_r_G* ^′0^ and Δ*_r_S* ^′0^ directly from conventional thermodynamic properties of species without the need of the complex computations required by the use of the Legendre transformed thermodynamic properties [6]. One of the advantages of this approach is, for instance, that it does not require the use of Δ*_f_H* ^′0^ and Δ*_f_G* ^′0^ data tables calculated for different values of pH and pMg [7].

Balanced biochemical reaction holds the same features of chemical equations. Balanced biochemical equations are of general validity and can be used for any biochemical reaction. The only difference between a balanced biochemical reaction and a chemical reaction is that in the former the concentration of H^+^ and Mg^2+^ are kept constant. This is the reason why the stoichiometric coefficients of the balanced biochemical reaction are functions of pH and pMg.

### Minimum value of G and G′ at equilibrium

It is a common belief that the Legendre transformed *G*′, *H*′ and *S*′ are the only thermodynamic properties appropriate to deal with biochemical reactions occurring at constant pH and pMg and that the transformed Gibbs energy *G*′, and not *G*, is minimized at equilibrium [3]. This opinion originates from the consideration that the biochemical reaction occurs in an open system with a change of the quantity of H^+^ and Mg^2+^ ions. The reaction can be exemplified as occurring in a reaction chamber connected to reservoirs with constant [H^+^] and [Mg^2+^] through semi-permeable membranes. Given that, hydrogen and magnesium are not conserved in the reaction chamber and this explains the reason why biochemical reactions are written in terms of sums of species [3]. As a consequence, according to this view, when the pH and pMg are specified only the transformed Gibbs energy *G*′ and not *G* provides the means for determining whether the reactions will go to the right or the left [5].

We recently showed that Δ*_r_G*′ of a biochemical reaction is equal to Δ*_r_G* of any single reaction involving selected chemical species of the biochemical system [8]. On this basis we hypothesize that also Δ*_r_G*′ of biochemical reaction and Δ*_r_G* of the corresponding balanced biochemical reaction, are the same at constant *T*, *P*, pH and pMg. In fact, a balanced biochemical reaction is equivalent to a chemical reaction and, as a consequence, it can be treated using the conventional chemical thermodynamics.

We calculated the change of *G* of the balanced biochemical reaction (9) and *G*′ of the biochemical reaction (3) as a function of the extent of reaction ξ at constant *T*, *P*, pH and pMg. We recall that the values of *G* and *G*′ refer to the standard Gibbs energy of formation of elements taken as zero.

**Figure 2:**
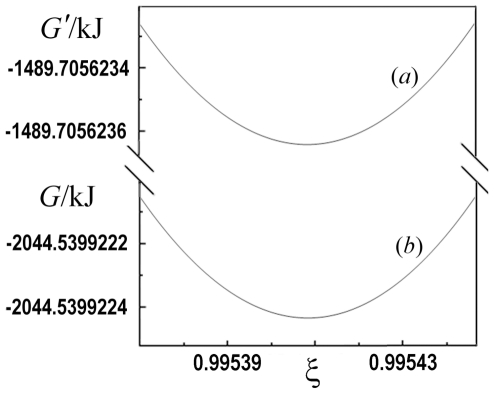
Plot of *G*′ (*a*) and *G* (*b*) as function of the extent of reaction ξ.

The initial state is a solution with a volume *V* ^0^ = 1 dm^3^, containing 1 mol of G6P at concentration *c* ^0^. The final state is a solution with a volume 2*V* ^0^ = 2 dm^3^, containing 1 mol of Glu, 1 mol of Pi both at concenntration *c* ^0^/2. In the course of reaction pH and pMg are constant while the volume *V* of the solution changes: *V* = (1+ξ)*V* ^0^. *V* ^0^, being equal to 1, will be omitted in the following equations. *G*′ of the biochemical reaction (3) is
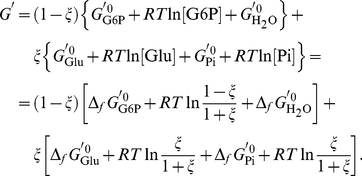
(21)The plot of *G*′ as a function of the extent of reaction ξ is reported in Fig. 2 (plot *a*) and shows a minimum at the value of ξ = 0.995412. The equilibrium constant expressed as a function of ξ is
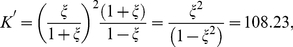
(22)giving Δ*_r_G* ^′0^ = −11.61 kJ mol^−1^.

*G* of the balanced biochemical reaction (9) is
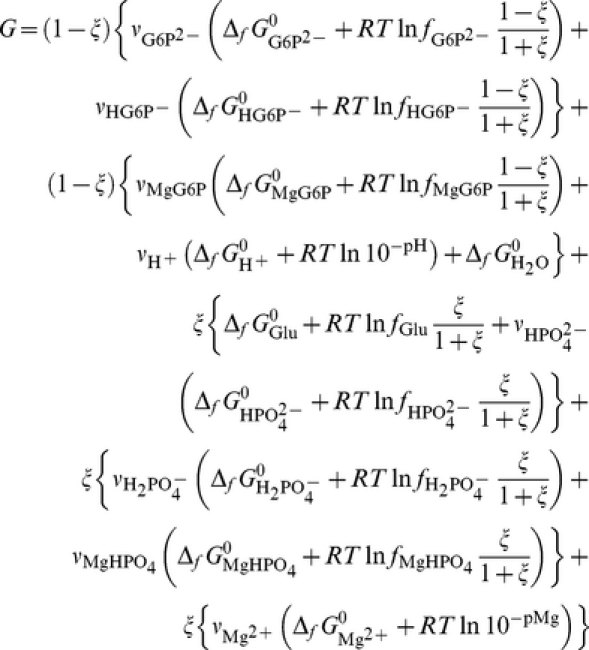
(23)


The plot of *G* as function of the extent of reaction ξ is reported in Fig. 2 (plot *b*) and shows that the minimum also occurs at ξ = 0.995412.

These results show that both *G* and *G*′ have the minimum at the same value of ξ. The difference *G*−*G*′ has a constant value of −554.83 kJ mol^−1^ in the whole range of ξ.

In agreement with Alberty [3], this difference is given by
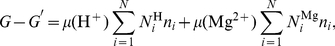
(24)where 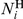 and 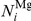 are the number of hydrogen and magnesium atoms in species *i* and *n_i_* is the amount of *i*. According to Alberty the number of species *N* would not include H^+^ and Mg^2+^ and, as a consequence, the difference *G−G*′ would not be constant in the course of reaction. On the contrary, using the stoichiometric coefficients (*n_i_* = ν*_i_*) of the balanced biochemical reaction (9), which includes the species H^+^ and Mg^2+^, we exactly obtain the constant value of −554.83 kJ mol^−1^ in the whole range of ξ. This result contradicts the established opinions that: *i*) the Legendre transformed *G*′ only is the appropriate thermodynamic function to deal with biochemical reactions occurring at specified pH and pMg, *ii*) the transformed Gibbs energy *G*′ and not *G* is minimized at equilibrium because the system exchanges H^+^ ed Mg^2+^. We show indeed that employing the balanced biochemical reaction there is no need of the Legendre transformed potential *G*′ to properly describe the thermodynamic state of a biochemical system, *G* being fully adequate to this scope.

### Conclusions

**Figure 3:**
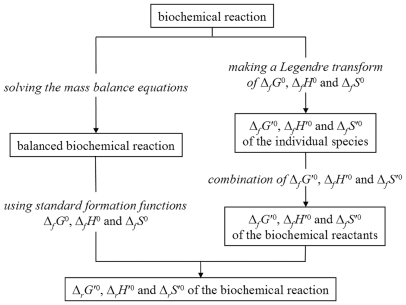
Flow diagram showing the different approaches to obtain the transformed thermodynamic properties: balanced biochemical reaction (left) and transformed formation functions (right).

The balanced biochemical equation allows a novel and simpler approach to the thermodynamics of the biochemical reactions. Indeed, the balanced biochemical equation is conceptually simpler as it just requires the knowledge of classical thermodynamics with no need to be acquainted to Legendre transforms thermodynamics, which in this case represents an un-necessary complication. In fact, as shown in Fig. 3, the balanced biochemical reaction approach entails less calculation steps compared to the Legendre transforms approach. The most elaborate procedure of the balanced biochemical reaction approach is the calculation of equilibrium concentrations of the different species; nevertheless this step is also required in the Legendre transforms approach for the calculation of Δ*_f_H* ^′0^.

In addition, using the balanced biochemical reaction approach, the calculations needed to obtain Δ*_r_N*(H^+^) and Δ*_r_N* (Mg^2+^) are trivial, while the Legendre transforms procedure [15] entails the following complex calculations:
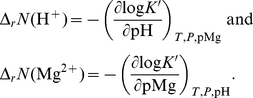
(25)


The quantities of the species entering and exiting the system are specified in the balanced biochemical reaction. This is the reason why Δ*_r_G* ^′0^, Δ*_r_H* ^′0^ and Δ*_r_S* ^′0^ can be directly calculated from the conventional standard formation thermodynamic properties of the species taking part to the biochemical reaction. It must be recalled that the value of Δ*_r_G* ^′0^ is obtained by the conditional constant *K* ^′^ (Δ*_r_G* ^′0^ = −*RT* ln*K*′) also known as apparent constant [2]. Coherently to the results of this study, where Δ*_r_G* ^′0^, Δ*_r_H* ^′0^ and Δ*_r_S* ^′0^ are obtained without using the Legendre transforms, we propose to name these thermodynamics properties “conditional” rather than “transformed”.

Finally, we also showed that the thermodynamic potential *G* is fully adequate to deal with biochemical reactions. The use of the transformed Gibbs energy *G*′ is correct, but unnecessary, and *G* is indeed minimized at equilibrium and therefore can properly give a criterion of spontaneous chemical change for a biochemical reaction. The introduction of the concept of the balanced biochemical reaction greatly simplifies the approach to the thermodynamics of complex systems, as the biochemical reactions are, making avoidable the use of the Legendre transforms. The biochemical balanced equations shows the possibility to deal the thermodynamics of biochemical reaction using the conventional thermodynamic properties.

In conclusion, the simple procedure of balancing the biochemical reactions is of general validity and makes possible to use the same approach when dealing with biochemical and chemical reactions. This allows the re-unification of the two worlds of chemical and biochemical thermodynamics, so far treated separately, within the same thermodynamic framework. Maybe, the balanced biochemical reaction can be regarded as the egg of Columbus for a notably easier approach to the biochemical thermodynamics.

## Supporting Information

Supporting Information S1 **This section describes in detail the procedure of balancing the biochemical reaction of the G6P hydrolysis and the calculation of the standard conditional properties Δ** ***_r_H*** **^′0^ and Δ** ***_r_G*** **^′0^ and of the changes in binding Δ** ***_r_N*** **(H^+^) and Δ** ***_r_N*** **(Mg^2+^).**
(DOC)
