# Supplementary material for: Balanced Biochemical Reactions: A New Approach to Unify Chemical and Biochemical Thermodynamics
Source: PLoS One. 2012 Jan 11;7(1):e29529. doi: 10.1371/journal.pone.0029529 (PMC3256155; doi:10.1371/journal.pone.0029529)

**Supporting Information**

**Calculation of stoichiometric coefficients of the balanced biochemical reaction of glucose-6-phosphate hydrolysis**: Step 1 of the procedure for balancing the biochemical reaction is based on the calculation of the concentration of free and complex species in a 1 M solution of the biochemical reactants at specified pH and pMg. Almost all biochemical reactions have complex species that are mononuclear with respect to each reactant. In this case the mass balance equation of each biochemical reactant is easily solved using binding polynomials and the free and complex species concentration are obtained [1]. Otherwise the mass balance equations can be solved using any of the available computer speciation programs, like Hyss [2]. The calculation procedure using binding polynomials is shown hereafter. The values of Δ*_f_G*0 used in the calculation are reported in Table S1-1.

The biochemical reaction is

G6P + H_2_O = Glu + Pi. (S1-1)

The mass balance equations (step 1) for the biochemical reactants are:

[$\text{G6P}$] = [$\text{G6P}^{2-}$] + [$\text{HG6P}^{-}$] + [$\text{MgG6P}$] =

[$\text{G6P}^{2-}$] + $K_{\text{HG6P}^{-}}$[$\text{G6P}^{2-}$][$\text{H}^{+}$] + $K_{\text{MgG6P}}$[$\text{G6P}^{2-}$][$\text{Mg}^{2+}$] =

(1 + $K_{\text{HG6P}^{-}}$[$\text{H}^{+}$] + $K_{\text{MgG6P}}$ [$\text{Mg}^{2+}$]) [$\text{G6P}^{2-}$] =

*B*_G6P_[$\text{G6P}^{2-}$], (S1-2)

[$\text{Pi}$] = [$\text{HPO}_{4}^{2-}$] + [$\text{H}_{2}\text{PO}_{4}^{-}$] + [$\text{MgHPO}_{4}$] =

[$\text{HPO}_{4}^{2-}$] + $K_{\text{H}_{2}\text{PO}_{4}^{-}}$[$\text{HPO}_{4}^{2-}$][$\text{H}^{+}$] + $K_{\text{MgHPO}_{4}}$[$\text{HPO}_{4}^{2-}$][$\text{Mg}^{2+}$] =

(1 + $K_{\text{H}_{2}\text{PO}_{4}^{-}}$[$\text{H}^{+}$] + $K_{\text{MgHPO}_{4}}$[$\text{Mg}^{2+}$]) [$\text{HPO}_{4}^{2-}$] =

*B*_Pi_[$\text{HPO}_{4}^{2-}$], (S1-3)

where the values of the binding polynomials

*B*_G6P_ = 1 + $K_{\text{HG6P}^{-}}$[$\text{H}^{+}$] + $K_{\text{MgG6P}}$ [$\text{Mg}^{2+}$], (S1-4)

*B*_Pi_ = 1 + $K_{\text{H}_{2}\text{PO}_{4}^{-}}$[$\text{H}^{+}$] + $K_{\text{MgHPO}_{4}}$[$\text{Mg}^{2+}$] (S1-5)

depend on [H^+^] and [Mg^2+^].

The values of the formation constants (*P* = 1 atm, *T* = 298.15 K, *I* = 0.25 M) are obtained from the $\Delta_{\text{r}}G^{0}$ of the reactions of formation of the complex species from the free species. For instance, for the reaction of formation of the complex species $\text{HG6P}^{-}$

$$\text{G6P}^{2-}\text{+ H}^{+}\text{ = HG6P}^{-}$$

we obtain:

$\Delta_{r}G^{0}\text{ = }\Delta_{f}G_{\text{HG6P}^{-}}^{0}-\Delta_{f}G_{\text{G6P}^{2-}}^{0}-\Delta_{f}G_{\text{H}^{+}}^{0}$= −1,801.40 + 1,767.18 + 0.81 = –33.41 kJ mol^-1^, (S1-6)

$K_{\text{HG6P}^{-}}=\text{exp}\left(-\Delta_{\text{r}}G^{0}/RT\right)=\text{exp}\left(33.41/2.47897\right)=7.135\cdot10^{5}$. (S1-7)

The values of the formation constants of the complex species herewith calculated are reported in Table S1‑2.

The values of the binding poynomials, at pH = 7 and pMg = 3, are obtained using (S1-4, S1-5):

*B*_G6P_ = 1.10053, *B*_Pi_ = 1.48484. (S1-8)

Using Eqs. (S1-2, S1-3) and taking [G6P] = [Pi] = 1 M, the concentrations of the free species G6P^2-^ and HPO_4_2-, are calculated:

[G6P^2-^] = 1/*B*_G6P_ = 1/1.10053 = 0.90866 M, [HPO_4_2-] = 1/*B*_Pi_ = 1/1.48484 = 0.67347 M. (S1-9).

Then, using the formation constants of the complex species (Table S2-2), the concentrations of HG6P^‑^, MgG6P, $\text{H}_{2}\text{PO}_{4}^{-}$ and MgHPO_4_ are obtained:

$\text{[HG6P}^{-}\text{]}$= $K_{\text{HG6P}^{-}}$×[H^+^]×[G6P^2-^] = 7.135·10^5^×10^-7^×0.90866 = 0.06483 M, (S1-10)

[MgG6P] = *K*_MgG6P_×[Mg^2+^]×[G6P^2-^] = 29.18×10^-3^×0.90866 = 0.02651 M, (S1-11)

$\text{[H}_{2}\text{PO}_{4}^{-}\text{]}$ = $K_{\text{H}_{2}\text{PO}_{4}^{-}}$×[H^+^]×[$\text{HPO}_{4}^{2-}$] = 4.472·10^6^×10^-7^×0.67347 = 0.30119 M, (S1-12)

[MgHPO_4_] = *K*_MgHPO4_×[Mg^2+^]×[$\text{HPO}_{4}^{2-}$] = 37.62×10^-3^×0.67347 = 0.02534 M. (S1-13)

In step 2, the stoichiometric coefficient of each species is obtained multiplying its own concentration by the stoichiometric coefficient of the corresponding biochemical reactant which, in reaction (S1-1), is equal to 1 for all the biochemical reactants:

reactants: $\nu_{\text{G6P}^{2-}}=0.90\text{866, }\nu_{\text{HG6P}^{-}}=0.06483,\text{ }\nu_{\text{MgG6P}}=0.02651,\text{ }\nu_{\text{H}_{2}\text{O}}=1$,

products: $\nu_{\text{Glu}}=1\text{, }\nu_{\text{HPO}_{4}^{2-}}=0\text{.67347, }\nu_{\text{H}_{2}\text{PO}_{4}^{-}}=0.30119\text{, }\nu_{\text{MgHPO}_{4}}=0.0\text{2534 }$.

In step 3 the Mg and H atoms have to be balanced. Taking the stoichiometric coefficients negative for reactants and positive for products and indicating with $N_{i}^{\text{Mg}}$and $N_{i}^{\text{H}}$ the number of atoms of Mg and H contained in species *i*, the Mg^2+^ and H^+^ stoichiometric coefficients are:

$\nu_{\text{Mg}^{\text{2+}}}=-\sum_{i} \nu_{i}N_{i}^{\text{Mg}}=0.00118$, $\nu_{\text{H}^{\text{+}}}=-\sum_{i} \nu_{i}N_{i}^{\text{H}}=-0.23636$. (S1-14)

0.00118 Mg^2+^ ions must be added to the right term of the reaction and 0.23636 H^+^ ions to the left term. The balanced biochemical reaction (*T* = 298.15 K, *I* = 0.25 M, pH = 7, pMg = 3) is:

0.90866 G6P^2-^ + 0.06483 HG6P^-^ + 0.02651 MgG6P + 0.23636 H^+^ + H_2_O =

Glu + 0.67347 $\text{HPO}_{4}^{2-}$ + 0.30119 $\text{H}_{2}\text{PO}_{4}^{-}$ + 0.02534 MgHPO_4_ + 0.00118 Mg^2+^. (S1-15)

If the equation is balanced, then the total ionic charges of left and right terms must be equal. In effect

$\sum_{r} \nu_{r}z_{r}=\sum_{p} \nu_{p}z_{p}=-1.64578$. (S1-16)

where *z* is the ionic charge of the species, indexes *r* and *p* refer to reagents and products respectively and stoichiomertic coefficients ν*_r_* and ν*_p_* are positive.

All these calculations can be easily formulated in a Microsoft Excel spreadsheet (Fig. S1-1). The input data are: pH (cell B6), pMg (cell B7), *I* (cell B8), and *T* (cell B9). The concentration values of G6P, Glu and Pi employed in the calculation of *Q'* and Δ*_r_G'* are reported in cells B10-B12.

The starting data are the values of Δ*_f_G*^0^ (cells B16-B25) and of Δ*_f_H*^0^ (cells C16-C25) of all the pseudoisomers (the species constituting the biochemical reactants) at *T* = 298.15 K and *I* = 0 M [3].

The values of Δ*_f_H*^0^, in a limited temperature range (298 ± 15 K), are essentially unchanged. The values of Δ*_f_G*^0^, for different temperatures are calculated by Van’t Hoff equation (cells F16-F25). The values of Δ*_f_G*^0^, for different ionic strength are calculated by the extended Debye-Huckel equation [4] and reported in cells G16-G25. The Δ*_r_G*0 of formation of the complex species and the corresponding equilibrium constants *K* are reported in cells J22-K25. The binding polynomials *B*_G6P_ e *B*_Pi_, obtained in cells K28 and K29 using the values of H^+^ and Mg^2+^ concentration shown in cells I10 and I11 respectively, allow to calculate the concentrations of free and complex species (fractional populations) reported in cells B28-B33. The stoichiometric coefficients of all reactants of the balanced biochemical reaction are finally obtained in cells C28-C37.

**Calculation of the conditional thermodynamics properties Δ*_r_G*′ 0, Δ*_r_H*′ 0 and of the change in binding Δ*_r_N*(H^+^) e Δ*_r_N*(Mg^2+^) of the balanced biochemical reaction of glucose-6-phospahate hydrolysis**: The values of Δ*_r_G*′ 0, Δ*_r_H*′ 0, Δ*_r_N*(H^+^) and Δ*_r_N*(Mg^2+^) of the balanced biochemical reaction of glucose-6-phospahate hydrolysis at different temperature and ionic strength values are calculated in the same spreadsheet where the stoichiometric coefficients of the balanced biochemical reaction are obtained. The products νΔ*_f_H*^0^ are carried out in cells D28-D37. Δ*_r_H*′ 0 is reported in cell E7. The $\Delta_{f}G^{'}$ of the reactants of the balanced biochemical reaction are calculated by Eqs. (11-14) and reported in cells F28-F37. These values are multiplied by the corresponding stoichiometric coefficients and reported in cells G28-G37. The sum of these quantities gives the value of Δ*_r_G*′ 0 which is reported in cell E6. The values of Δ*_r_N*(H^+^) and Δ*_r_N*(Mg^2+^), reported in cells E8 and E9, are the stoichiometric coefficients of H^+^ and Mg^2+^ (cells C36 and C37) changed in sign.

The value of the quotient of reaction *Q'*, calculated using the concentrations values of B10-B12 cells, is reported in cell E10. The corresponding value of Δ*_r_G'* is reported in cell E11.

We made available the Excel file at: <http://www.albertohyp.it/RM2/G6P&ATP_hydrolysis.xls>

which can be used to perform the above calculations at different pH, pMg and biochemical reactant concentrations for both G6P and ATP hydrolysis reactions.

**References**

1. Iotti S, Sabatini A, Vacca A (2010) Chemical and biochemical thermodynamics: from ATP hydrolysis to a general reassessment. Journal of Physical Chemistry B 114: 1985-1993.

2. Alderighi L, Gans P, Ienco A, Daniel P, Sabatini A, et al. (1999) Hyperquad simulation and speciation (HySS): a utility program for the investigation of equilibria involving soluble and partially soluble species. Coordination Chemistry Reviews 184: 311-318.

3. Alberty RA (1992) Calculation of transformed thermodynamic properties of biochemical reactants at specified pH and pMg. Biophysical Chemistry 43: 239-254.

4. Alberty RA (2003) Thermodynamics of the Hydrolysis of Adenosine Triphosphate as a Function of Temperature, pH, pMg, and Ionic Strength. Journal of Physical Chemistry B 107: 12324-12330.

LEGEND TO FIGURE

**Figure S1-1.** Electronic sheet containing the calculations of the stoichiometric coefficients, of the thermodynamics properties Δ*_r_G*′ 0, Δ*_r_H*′ 0 and of the change in binding Δ*_r_N*(H^+^) , Δ*_r_N*(Mg^2+^) of the balanced biochemical reaction of glucose-6-phospahate hydrolysis

**TABLE S1-1: Standard Gibbs energies and enthalpies of formation of free and complex species at 298.15 K and I = 0.25 M ^a^**

| Species | Δ*_f_G*^0^, kJ mol^-1^ | Δ_f_H^0^, kJ mol^-1^ |
| --- | --- | --- |
| $\text{G6P}^{2-}$ | -1,767.18 | -2,274.80 |
| $\text{HG6P}^{-}$ | -1,801.40 | -2,274.23 |
| $\text{MgG6P}$ | -2,234.08 | -2,732.04 |
| $\text{Glucose}$ | -915.90 | -1,262.19 |
| $\text{HPO}_{4}^{2-}$ | -1,099.34 | -1,297.36 |
| $\text{H}_{2}\text{PO}_{4}^{-}$ | -1,138.11 | -1,302.19 |
| $\text{MgHPO}_{4}$ | -1,566.87 | -1,753.80 |
| $\text{H}_{2}\text{O}$ | -237.19 | -285.83 |
| $\text{H}^{+}$ | -0.81 | 0.41 |
| $\text{Mg}^{2+}$ | -458.54 | -465.36 |

^a^ The values reported in the table are taken from Ref. [3].

**TABLE S1-2: Standard Gibbs energies of reaction and formation constants of complex species at 298.15 K and I = 0.25 M**

| Reaction | Δ*_r_G*^0^, kJ mol^-1 a^ | K |
| --- | --- | --- |
| $\text{HPO}_{4}^{2-}+\text{ H}^{+}=\text{ H}_{2}\text{PO}_{4}^{-}$ | –37.96 | 4.470·10^6^ |
| $\text{HPO}_{4}^{2-}+\text{ Mg}^{2+}=\text{ MgHPO}_{4}$ | –8.99 | 37.58 |
| $\text{G6P}^{2-}+\text{ H}^{+}\text{ }=\text{ HG6P}^{-}$ | –33.41 | 7.131·10^5^ |
| $\text{G6P}^{2-}+\text{ Mg}^{2+}\text{ }=\text{ MgG6P}$ | –8.36 | 29.15 |

^a^ Calculated using the Δ*_f_G*^0^ values reported in Table 1.

LEGEND TO FIGURE

**Figure S1-1. Microsoft Excel spreadsheet for the calculations of thermodynamics properties of glucose-6-phosphate hydrolysis.**


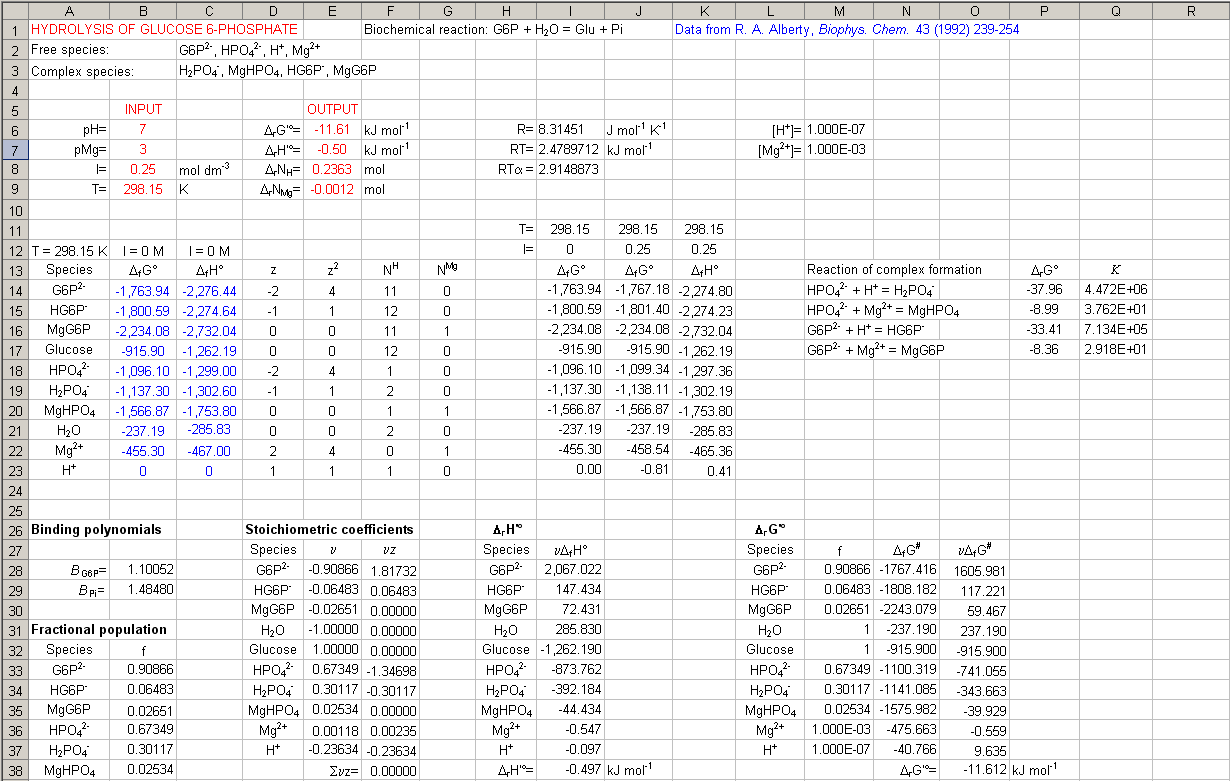

Supplement: Supporting Information S1 — This section describes in detail the procedure of balancing the biochemical reaction of the G6P hydrolysis and the calculation of the standard conditional properties Δ rH ′0 and Δ rG ′0 and of the changes in binding Δ rN (H+) and Δ rN (Mg2+). (DOC) [file pone.0029529.s001.doc]
